# Cognitive function after electroconvulsive therapy for depression: relationship to clinical response

**DOI:** 10.1017/S0033291720000379

**Published:** 2021-07

**Authors:** Ian M. Anderson, R. Hamish McAllister-Williams, Darragh Downey, Rebecca Elliott, Colleen Loo

**Affiliations:** 1 Neuroscience and Psychiatry Unit, University of Manchester, Manchester Academic Health Science Centre, Manchester, UK; 2 Newcastle University, Cumbria, Northumberland Tyne and Wear NHS Foundation Trust, Newcastle upon Tyne, UK; 3 Faculty of Biology, Medicine and Health, University of Manchester, Manchester, UK; 4 University of New South Wales, Black Dog Institute & St George Hospital, Sydney, Australia

**Keywords:** Cognition, depressive disorder, electroconvulsive therapy, memory, remission

## Abstract

**Background:**

As uncertainty remains about whether clinical response influences cognitive function after electroconvulsive therapy (ECT) for depression, we examined the effect of remission status on cognitive function in depressed patients 4 months after a course of ECT.

**Method:**

A secondary analysis was undertaken on participants completing a randomised controlled trial of ketamine augmentation of ECT for depression who were categorised by remission status (MADRS ⩽10 *v.* >10) 4 months after ECT. Cognition was assessed with self-rated memory and neuropsychological tests of anterograde verbal and visual memory, autobiographical memory, verbal fluency and working memory. Patients were assessed through the study, healthy controls on a single occasion, and compared using analysis of variance.

**Results:**

At 4-month follow-up, remitted patients (*N* = 18) had a mean MADRS depression score of 3.8 (95% CI 2.2–5.4) compared with 27.2 (23.0–31.5) in non-remitted patients (*N* = 19), with no significant baseline differences between the two groups. Patients were impaired on all cognitive measures at baseline. There was no deterioration, with some measures improving, 4-months after ECT, at which time remitted patients had significantly improved self-rated memory, anterograde verbal memory and category verbal fluency compared with those remaining depressed. Self-rated memory correlated with category fluency and autobiographical memory at follow-up.

**Conclusions:**

We found no evidence of persistent impairment of cognition after ECT. Achieving remission improved subjective memory and verbal memory recall, but other aspects of cognitive function were not influenced by remission status. Self-rated memory may be useful to monitor the effects of ECT on longer-term memory.

Cognitive impairment is a recognised short-term consequence of electroconvulsive therapy (ECT) (Semkovska & McLoughlin, 2010) but controversy remains about whether or not there is a persistent longer-term impairment (Read, Cunliffe, Jauhar, & McLoughlin, 2019; Rose, Fleischmann, Wykes, Leese, & Bindman, 2003). Critics of ECT emphasise the subjective experience of patients surveyed after ECT with studies finding that 29–55% of the respondents reported persistent or permanent memory loss attributed to ECT (Rose et al., 2003). The perceived potential for cognitive adverse effects is seen as a limiting factor in the use of ECT (Finnegan & McLoughlin, 2019).

In apparent contrast to retrospective reports, a recent systematic review of subjective memory measured prospectively found that most studies reported an improvement in memory after ECT, but noted considerable heterogeneity in both the results and in how subjective memory was assessed (Vann & McCollum, 2019). A systematic review found that the performance on many objective neuropsychological tests is transiently impaired by ECT but improves to baseline or better within a few weeks after completing treatment (Semkovska & McLoughlin, 2010). More recent follow-up studies of assessing cognition a few months after ECT have also been consistent in finding no evidence of worsening, with some domains improving, compared with before ECT although the results conflict about which aspects of cognition show improvement (Bodnar et al., 2016; Bosboom & Deijen, 2006; Mohn & Rund, 2016; Nuninga et al., 2018; Obbels et al., 2018; Vasavada et al., 2017; Verwijk et al., 2014; Ziegelmayer et al., 2017). A remaining area of uncertainty however is whether or not past memories, especially autobiographical memory, might be adversely affected by ECT given the lack of evidence (Semkovska & McLoughlin, 2010) and methodological difficulties in the current assessment tools (Semkovska & McLoughlin, 2013). It is possible that negative subjective assessment of the effect of ECT could be contributed to by loss of past memories or impairments that are difficult to detect with standard tests (Finnegan & McLoughlin, 2019).

Depression is itself associated with widespread impairment in cognitive function (Pan et al., 2019) and a key issue in the assessment of cognition after ECT for depression is the degree to which mood state influences cognitive assessment results; low mood could potentially lead to the misattribution to ECT of memory and other cognitive problems that are in fact associated with persisting depression. A further complication is that when people recover from depression, they have cognitive impairment which is related to the number of previous episodes (Semkovska et al., 2019) so the experience of impaired cognitive function after recovery from depression may be due to prior depression rather than to the treatment received. A systematic review of subjective memory after ECT reported the improvement following ECT correlated with improved depression, although there was considerable heterogeneity between studies (Vann & McCollum, 2019). In contrast, those investigating objective tests of cognition have nearly all found no relationship between performance and mood (Bosboom & Deijen, 2006; Fernie, Bennett, Currie, Perrin, & Reid, 2014; Maric et al., 2016; Nuninga et al., 2018; Vasavada et al., 2017; Ziegelmayer et al., 2017). The reasons for this discrepancy are not known but it is recognised that subjective and objective measures of cognition often do not correlate (Finnegan & McLoughlin, 2019), and in addition, studies of cognitive test performance after ECT have been mostly statistically underpowered and only reported mood effects incidentally.

The aim of this study was to examine the effect of mood state on cognitive function following a course of ECT. In order to do this, we performed a secondary analysis of cognition over time in patients receiving ECT in a clinical trial, grouped according to their remission status 4 months after ECT. In addition, we compared cognition in patients to that of matched healthy controls. We hypothesised that remitted patients would have better subjective memory and objectively assessed cognition than those who had not remitted, but that they would remain impaired compared to healthy controls.

## Methods

### Participants

This is a secondary analysis of the Ketamine-ECT study not specified in the original trial protocol. Patients who completed the study were grouped according to whether or not they were in remission from depression at the final assessment 4 months after ECT. The Ketamine-ECT study was a UK multicentre, randomised, placebo-controlled trial of intravenous ketamine as an adjunct to ECT in severely ill depressed patients that has been previously reported (Anderson et al., 2017a, 2017b). Inclusion criteria were a diagnosis of a moderate or severe major depressive episode in unipolar or bipolar disorder by DSM-IV criteria (American Psychiatric Association, 2000) with ECT planned as part of clinical care, age ⩾18 years, ability to give valid consent with a verbal IQ equivalent to ⩾85 [Wechsler Test of Adult Reading, WTAR (Wechsler, 2001)], able to complete neuropsychological testing and medically fit to receive ketamine. Main exclusion criteria were ECT in the last 3 months, detention under the Mental Health Act (1983, amended 2007), a primary psychotic or schizoaffective disorder, current primary obsessive–compulsive disorder, anorexia nervosa, or history of drug or alcohol dependence (DSM-IV criteria), organic brain disease or significant medical illness affecting neuropsychological function, <24 on the Mini Mental State Examination (MMSE) (Folstein, Folstein, & McHugh, 1975), contraindication to ketamine, risk of pregnancy or breastfeeding. Healthy controls were recruited by advertisement and from relatives, and prospectively sex and age group matched with patients in the main study. They were required to be in good physical health with no history of personal, or first-degree family, psychiatric disorder, significant medical illness, psychotropic medication or other medication that could interfere with neuropsychological function or an MMSE <24.

The study complied with the Helsinki Declaration of 1975, as revised in 2008. Ethical approval was granted by the North West-Liverpool East Research Ethics Committee (Ref. 12/NW/0021) on 25 January 2012. Clinical Trial Authorisation was received from the UK Medicines and Healthcare products Regulatory Agency (23148/0004/001-0001). All participants gave written informed consent to participate. The study is registered with the International Standard Randomised Clinical Trial Number registry (ISRCTN14689382) and with the EU Clinical Trial register (EudraCT number 2011-005476-41).

### Study design and procedures

The Ketamine-ECT Study assessed whether ketamine given in addition to ECT would improve clinical outcomes, and found it had no effect on efficacy or cognitive function compared with saline (Anderson et al., 2017b). Following baseline assessment, depressed patients were randomised to intravenous ketamine 0.5 mg/kg or saline augmentation of their anaesthetic induction agents and received ECT twice weekly based on the standard clinical ECT protocols (Royal College of Psychiatrists, 2005). Electrode placement was predominantly bilateral (BL) with a minority receiving right unilateral (RUL) ECT using constant-current brief pulse (0.5 ms pulse width) stimuli to induce seizure with a treatment dose of 1.5 or 4–6 times seizure threshold for BL and RUL placement respectively, determined by stimulus titration in the first session treatment. The goal was to treat patients to remission (Montgomery–Åsberg Depression Rating Scale, MADRS ⩽10) (National Institute for Health and Care Excellence, 2009) but the decision to finish ECT treatment was taken by the treating clinical team.

Cognitive assessments in patients were carried out before ECT (baseline), after four ECT treatments, within 5 days after the end of ECT treatment (end of ECT), and 1 and 4 months after the end of ECT (1- and 4-month follow-up). Efficacy was assessed at baseline, weekly during the ECT course, at the end of ECT and at the follow-up appointments. Only results from baseline, end of ECT and the two follow-up appointments are reported in this study. Healthy controls were assessed on a single occasion.

### Assessments

The full range of assessments is given in the main study reports (Anderson et al., 2017a, 2017b) with those relevant presented here. Baseline assessment included the Mini International Neuropsychiatric Interview (Sheehan et al., 1998) to determine diagnosis, the Massachusetts General Hospital Scale (MGHS) (Fava, 2003) to record antidepressant drug treatment in the current episode, the WTAR (Wechsler, 2001) as a measure of premorbid intellectual functioning (IQ) and the MMSE (Folstein et al., 1975) to screen for cognitive impairment. The key efficacy assessment was depression severity assessed by the observer-rated MADRS (Montgomery & Asberg, 1979).

The neuropsychological assessment consisted of tests involving verbal and visual memory, attention and verbal fluency/executive function (Anderson et al., 2017a, 2017b) with the key outcomes reported here.

#### Subjective memory

Self-reported Global Self Evaluation of Memory (GSE-My) (Berman, Prudic, Brakemeier, Olfson, & Sackeim, 2008) assessed subjective global memory and the participants' belief about how their memory had been affected by ECT, rated using a seven-point bipolar scale (extremely bad/negative to extremely good/positive). At baseline, participants were asked to rate their expectation about how ECT would affect their memory with this time-point taken as no effect for the analysis over time of their belief about how ECT had affected their memory. Expectation and belief about ECT's effect on memory were also dichotomised as worse or no change/better as there were very few endorsements of improvement.

#### Anterograde memory and retrograde autobiographical memory

Anterograde verbal memory was measured by delayed recall on the Hopkins Verbal Learning Test – Revised (HVLT-R-DR) (Benedict, Schretlen, Groninger, & Brandt, 1998), which involves learning a list of 12 words and being asked to recall as many words as possible following an interval of 30 min. This provides a composite test of learning, retention and ability to access the previously learnt words. Different versions of six alternate forms were used at each time-point in two orders of presentation.

Anterograde visual memory was assessed using delayed reproduction from the Medical College of Georgia Complex Figure Test (MCGCFT) (Meador et al., 1993). The task consists of copying a complex line drawing with multiple elements and then reproducing it from memory, immediately and after a 30 min delay. This tests visuospatial learning, retention, retrieval and executive planning; four alternate forms which were used at different time-points in two orders.

The Columbia Autobiographical Memory Interview – short form (AMI-SF) (McElhiney, Moody, & Sackeim, 1997) was used to assess memory for personal past events; an initial interview elicits personal memories covering six areas which are then asked about on subsequent occasions and rated according to consistency (percentage correct of the initial information provided). Improvement over baseline cannot be measured because new or more detailed information is not scored. A normal decline in scores over time in both healthy participants and depressed patients not receiving ECT has been reported using a revised scoring system, but normative data are lacking for the original test (Semkovska & McLoughlin, 2013).

#### Verbal fluency and working memory

The Controlled Oral Word Association Test (COWAT) (Benton, Hamsher, & Sivan, 1994) tested letter and category fluency. Task performance is related to the ability to update information, a function of executive control, and verbal ability, in particular lexical access speed for category fluency (Shao, Janse, Visser, & Meyer, 2014). Participants were asked to generate as many words as they could in 1 min for words starting with letters (F, A, S) and in a given category (animals or fruit and vegetables). The same letters were presented on subsequent tests and the two categories were alternated in two different orders.

Digit span backwards (Wechsler, 1981) involves strings of digits of increasing length being presented in a standard way and the participant repeating them in reverse order. The maximum number of digits remembered correctly is reported and is a test of attention, working memory storage and manipulation.

### Analysis

Data were analysed using IBM SPSS Statistics Version 22 (http://www.IBM.com). At 4-month follow-up, patients were divided into two groups categorised as being in remission (MADRS ⩽10) or non-remitted (MADRS >10). Two patients, one in each group, had missing data at one intermediate time-point and these were interpolated from adjacent values. Comparisons between healthy controls and the two groups of depressed patients were by one-way analysis of variance (ANOVA) (with Bonferroni correction for post-hoc tests), *t* tests, χ^2^ and Mann–Whitney *U* tests as applicable for parametric, non-parametric and categorical comparisons. Analysis of patients over time was by two-way repeated-measures ANOVA with factors for time (assessment point) and group (remitted, non-remitted). A drug factor (ketamine, saline) was not included given the lack of significant effect on cognitive outcome (Anderson et al., 2017b). Post-hoc testing was by simple contrasts for (a) the main effect of time in all patients, and (b) the time × group interaction to determine differential effects over time depending on the remission group. Significance level was taken as *p* < 0.05 (Huyhn–Feldt corrected for the repeated-measures ANOVA). An exploratory correlational analysis (Spearman's *ρ*) at 4-month follow-up was used to investigate the association of subjective memory with depression severity and task performance given the reports of their association in the literature (Berman et al., 2008; Vann & McCollum, 2019). Data are presented as mean and 95% confidence interval (95% CI), or median and interquartile range (IQR). The assessment time-points are taken as the median time in weeks since the baseline assessment for all patients (6, 10, 22 weeks), as values for each patient varied according to the number of ECT treatments received.

## Results

**Fig. 1.**
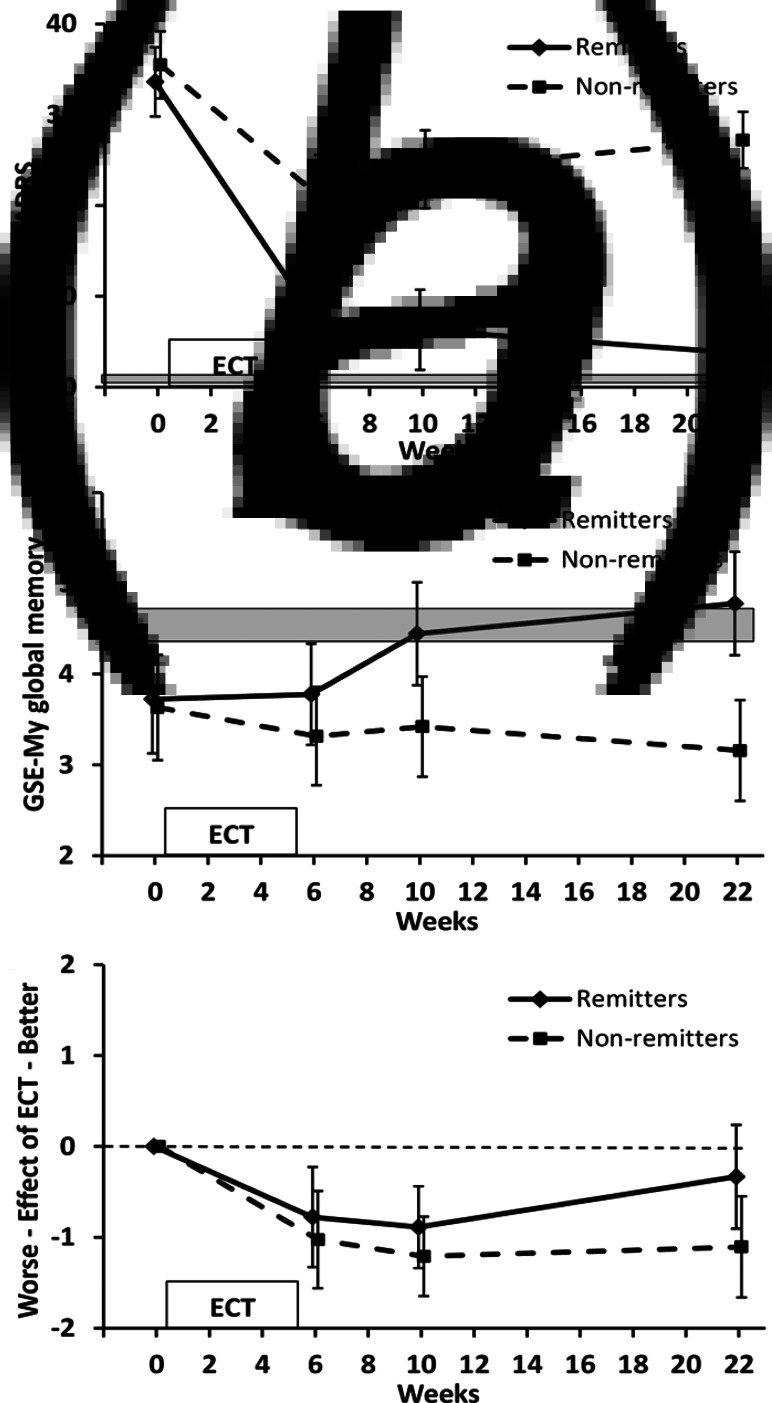
MADRS and GSE-My ratings for the current memory and the effect of ECT over time. Values are mean and 95%CI. Shaded area indicates 95% CI range for healthy controls. (*a*) MADRS scores for patient groups illustrated based on the presence or absence of remission at 4-month follow-up. (*b*) GSE-My current memory. ANOVA group × time *p* = 0.02; group × time contrast between baseline and 4-month follow-up *p* = 0.001. (*c*) GSE-My effect of ECT. ANOVA time *p* < 0.001, time contrasts between baseline and subsequent time-points *p* ⩽ 0.001.

Seventy-nine patients were randomised with the 37 patients who were assessed 4 months after the end of ECT (18 remitted, 19 non-remitted at final assessment) included in this analysis, together with 56 healthy controls. Clinical and cognitive assessments over time in the intention-to-treat patient population and patient flow through the study are given in the main study report (Anderson et al., 2017b) with timing and reasons for dropping out summarised in online Supplementary Material. Patients who dropped out were broadly similar to those completing the study in their baseline characteristics and cognitive function (online Supplementary Table S1). There was a wide separation in depression scores at 4-month follow-up (Fig. 1*a*) with the highest MADRS score in the remitted group 9, and the lowest in the non-remitted group 16. Remitted patients still scored higher than the healthy controls (3.8, 95% CI 2.2–5.4 *v.* 0.8, 0.5–1.3, *p* = 0.045), while the non-remitted patients remained moderately to severely depressed (MADRS 27.2, 23.0–31.5, *p* < 0.001 *v.* healthy controls and remitted patients). The two patient groups did not differ significantly on demographic or illness-related measures at baseline (Table 1). Apart from one patient in the non-remitted group, all were on an antidepressant which was combined with an antipsychotic drug in just over half of patients. Most patients were taking a selective serotonin reuptake inhibitor or serotonin and noradrenaline reuptake inhibitor; combination antidepressant treatment (usually with mirtazapine) occurred in five patients in the remitted group and four in the non-remitted group.

**Table 1.** Comparison of healthy controls and patients at baseline grouped according to remission status at 4-month follow-up

Measure	Healthy controls (*N* = 56)	Remitted patients (*N* = 18)	Non-remitted patients (*N* = 19)	*p*^a^
	Mean (95% CI)^b^	Mean (95% CI)^b^	Mean (95% CI)^b^	
Age	56.3 (53.1–59.5)	57.5 (51.9–63.1)	50.4 (45.3–55.4)	0.11
Sex, F:M	34:22	14:4	9:10	0.16
IQ	111 (109–114)	108 (103–114)	107 (102–113)	0.27
Years in FT education	14.6 (13.7–15.5)	14.7 (12.7–16.6)	14.0 (12.2–15.8)	0.79
MMSE	29.8 (29.6–29.9)	28.7 (27.8–29.6)**	28.7 (27.9–29.6)**	**<0.001**
Diagnosis, UP:BP depression	–	17:1	15:4	0.17
Lifetime depressive episodes	–	5.9 (3.1–8.6)	6.7 (2.5–10.9)	0.72
Depression episode duration, months, Median [IQR]		7 [2 to 20]	11 [6 to 22]	0.11
MADRS	0.9 (0.5–1.3)	33.6 (29.6–37.6)***	35.5 (31.7–39.2)***	**<0.001**
MGH Treatment resistance, Median [IQR]	–	3.5 [2.4–4.1]	3.5 [2.5–5.0]	0.44
Previous ECT, Yes:No	–	8:10	11:8	0.41
Psychotropic medication, *N*	–			0.71
Any antidepressant		18	18	
(SSRI/SNRI/TCA/other AD)		(5/11/1/6)	(7/7/2/6)	(0.67)
Antipsychotic		11	9	
Lithium		4	4	
Antiepileptic drug		1	3	
Hypnotic/anxiolytic		5	9	
GSE-My Current global memory	4.5 (4.3–4.8)	3.7 (3.1–4.3)*	3.6 (3.0–4.2)**	**0.001**
GSE-My Expectation of ECT on memory, Negative:Nil/Positive	–	12:6	13:6	0.91
HVLT-R Delayed recall	8.9 (8.3–9.6)	6.5 (4.7–8.4)**	7.0 (5.8–8.2)*	**0.002**
MCGCFT Delayed recall	25.2 (23.6–26.7)	18.2 (14.6–21.8)***	20.3 (17.3–23.3)*	**<0.001**
AMI-SF Baseline score	–	42.8 (37.3–48.2)	48.0 (44.4–51.6)	0.10
COWAT Letter fluency	45.5 (42.4–48.7)	36.7 (30.3–43.2)*	35.7 (28.5–43.0)*	**0.004**
COWAT Category fluency	23.3 (22.1–24.5)	16.9 (14.3–19.5)***	17.1 (14.6–19.7)***	**<0.001**
Digit span backward	4.9 (4.5–5.2)	3.4 (3.0–3.9)***	3.7 (3.1–4.4)**	**<0.001**

AD, antidepressant drug; AMI-SF, Columbia Autobiographical Memory Interview-Short Form; BP, bipolar; COWAT, Controlled Oral Word Association Test; ECT, electroconvulsive therapy; FT, full-time; GSE-My, Global Self Evaluation of Memory; HVLT-R, Hopkins Verbal Learning Test-Revised; IQ, intelligence quotient; IQR, interquartile range; MADRS, Montgomery–Åsberg Depression Rating Scale; MCGCFT, Medical College of Georgia Complex Figure Test; MGH, Massachusetts General Hospital; SSRI, selective serotonin reuptake inhibitor; SNRI, serotonin and noradrenaline reuptake inhibitor; TCA, tricyclic antidepressant; UP, unipolar.

a One-way analysis of variance/*t* test/χ^2^/Mann–Whitney *U* test as applicable.

b Unless otherwise stated.

*p < 0.05, **p < 0.01, ***p < 0.001 *v*. healthy controls (Bonferroni corrected). No significant differences between patient groups.

Table 2 shows treatment-related variables. The only significant difference was that more patients in the remitted group had remitted by the end of ECT treatment than in the non-remitted group. Non-remitted patients received non-significantly more ECT treatments and hence had a slightly longer gap between baseline and end of ECT assessment. There was only a modest change in medication which did not differ between the groups.

**Table 2.** Treatment-related variables in remitted and non-remitted patients at 4-month follow-up

Measure	Remitted patients (*N* = 18)	Non-remitted patients (*N* = 19)	*p*^a^
	Mean (95% CI)^b^	Mean (95% CI)^b^	
Number of ECT treatments	10.1 (8.1–12.2)	11.6 (9.4–13.8)	0.31
BL:RUL ECT	17:1	16:3	0.32
Received ketamine:saline	7:11	12:7	0.14
In remission at end of ECT, Yes:No	13:5	7:12	**0.005**
Time between baseline and end of ECT assessment, weeks	5.4 (4.5–6.3)	6.8 (5.3–8.3)	*p* = 0.10
Time between last ECT and end of ECT assessment, days	3.8 (2.5–5.0)	3.8 (2.8–4.9)	*p* = 0.94
Psychotropic medication at FU, *N*			0.41
Any antidepressant	18	18	
(SSRI/SNRI/TCA/other AD)	(4/13/1/5)	(5/8/3/5)	(0.53)
Antipsychotic	13	11	
Lithium	3	6	
Antiepileptic drug	0	4	
Hypnotic/anxiolytic	6	10	

BL, bilateral electrode placement; ECT, electroconvulsive therapy; RUL, right unilateral electrode placement; SSRI, selective serotonin reuptake inhibitor; SNRI, serotonin and noradrenaline reuptake inhibitor; TCA, tricyclic antidepressant.

a *t* test/χ^2^ as applicable.

b Unless otherwise stated.

### Baseline comparisons of mood and cognition

At baseline, the patient groups did not differ significantly from each other or healthy controls in age, sex distribution, IQ or education (Table 1), although patients in the non-remitted group were a little younger with a higher proportion of men than the other groups. Patients had higher depression scores than healthy controls and had slightly lower MMSE scores, with impairment on all neuropsychological tests and poorer subjective memory. The two patient groups did not differ in depression severity or on cognitive measures and about two-thirds expected a negative effect of ECT on memory (see Table 1).

### Change in cognitive measures over time

#### Subjective memory assessment

GSE-My current memory scores (Fig. 1*b*) showed no significant effect of time (*F*_3,105_ = 1.363, *p* = 0.26) but a significant effect of group (*F*_1,35_ = 10.003, *p* = 0.003) and an interaction between group and time (*F*_3,105_ = 3.692, *p* = 0.02). Contrasts showed no significant effect of ECT (time contrast between baseline and end of ECT, *p* = 0.65) but a significant group × time interaction between baseline and 4-month follow-up (*p* = 0.001) and a trend between baseline and 1-month follow-up (*p* = 0.063). Non-remitted patients did not significantly change over time, whereas remitted patients showed improvement after ECT, with values at 1- and 4-month follow-up similar to healthy controls. In the remitted group, 2/18 rated their memory worse at 4-month follow-up than baseline compared with 10/19 in the non-remitted group (*p* = 0.02).

Patients' self-evaluation of the effect of ECT on the GSE-My (Fig. 1*c*) showed a significant effect of time (*F*_3,105_ = 10.868, *p* < 0.001) and a trend to a group effect (*F*_1,35_ = 2.928, *p* = 0.096) but no interaction between group and time (*F*_3,105_ = 1.300, *p* = 0.28). Overall patients reported a negative effect of ECT on memory which plateaued between 1- and 4-month follow-up in non-remitters but returned towards no effect in remitters. Time contrasts showed this negative evaluation was significant at all time-points compared to baseline for patients taken together (*p* ⩽ 0.001). There were no significant group × time contrasts in keeping with the overall result ANOVA (baseline compared with 4-month follow-up, *p* = 0.057) although the final value in remitters did not significantly differ from 0 (i.e. no effect of ECT, *p* = 0.3).

Of the 25 patients who expected a negative effect of ECT on memory before ECT, 9/13 (69%) non-remitters and 4/12 (33%) remitters reported a negative effect at follow-up; for those who expected no effect or a positive effect, the respective figures for a negative effect at follow-up were 3/6 (50%) and 2/6 (33%). The differences in proportions were not significant (*p* = 0.3).

#### Objective anterograde memory and retrograde biographical memory

**Fig. 2.**
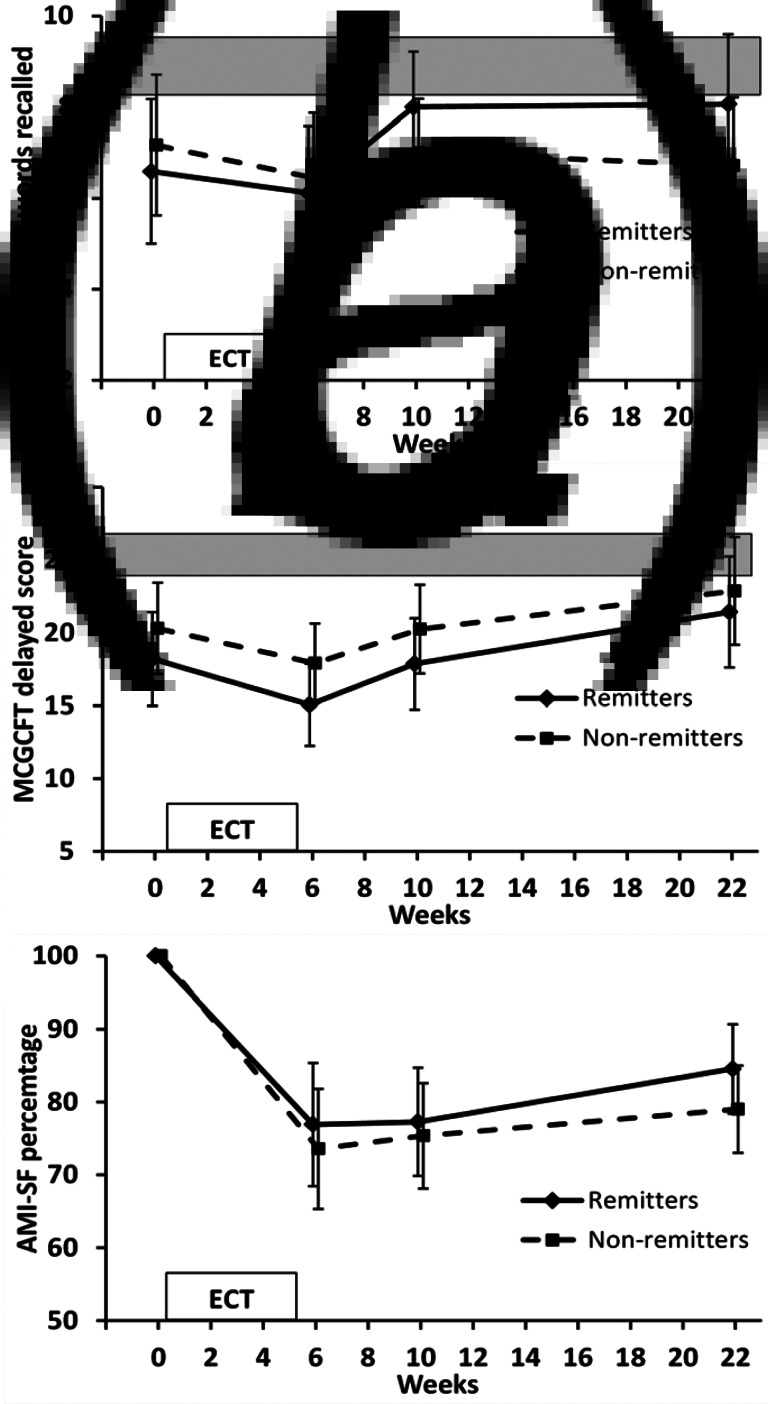
Anterograde verbal (HVLT-R-DR) and visual (MCGCFT) memory and retrograde autobiographical memory (AMI-SF) over time. Values are mean and 95% CI. Shaded area indicates 95% CI range for healthy controls. (*a*) HVLT-R-DR. ANOVA time *p* = 0.02; time contrast between the end of ECT and 4-month follow-up *p* = 0.02. Group × time contrast between baseline and 4-month follow-up *p* = 0.04. (*b*) MCGCFT. ANOVA time *p* < 0.001; time contrasts between baseline and end of ECT *p* = 0.005 and end of ECT and 4-month follow-up *p* < 0.001. (*c*) AMI-SF. ANOVA time *p* < 0.001; time contrasts between baseline and end of ECT *p* < 0.001 and end of ECT and 4-month follow-up *p* = 0.005.

Delayed verbal memory measured with the HVLT-R-DR (Fig. 2*a*) showed a significant effect of time (*F*_3,105_ = 3.425, *p* = 0.02) and a trend for a group × time interaction (*F*_3,105_ = 2.490, *p* = 0.065). Time contrasts showed that the slight decline in the number of words recalled between baseline and end of ECT was not significant (*p* = 0.21), but was followed by a significant improvement between the end of ECT and 4-month follow-up (*p* = 0.02). Remitters improved significantly compared to non-remitters between baseline and 4-month follow-up (time × group contrast *p* = 0.04) with a trend at 1-month follow-up (*p* = 0.07), and did not differ significantly from the healthy controls at both follow-up assessments (*p* > 0.5). Non-remitters remained impaired compared to healthy controls at 4-month follow-up (*p* < 0.01).

Delayed reproduction of a complex figure assessed with the MCGCFT (Fig. 2*b*) changed significantly with time (*F*_3,105_ = 13.082, *p* < 0.001) with no differential effect by group (group × time *F*_3,105_ = 0.205, *p* = 0.88). Time contrasts showed a significant decline in score after ECT compared with baseline (*p* = 0.005) and a subsequent improvement so that at 4-month follow-up, it was significantly better than baseline, end of ECT and 1-month follow-up (*p* ⩽ 0.007) and did not differ significantly from healthy controls for either group (*p* ⩾ 0.12).

Retrograde autobiographical memory consistency measured by the AMI-SF (Fig. 2*c*) changed significantly with time (*F*_3,105_ = 46.693, *p* < 0.001) with a nadir at the end of ECT and no differential effect by group (group × time *F*_3,105_ = 0.482, *p* = 0.69). Time contrasts showed consistency was significantly lower than 100% at all subsequent time-points (*p* < 0.001) as expected, but significantly increased from the end of ECT to 4-month follow-up (*p* = 0.005) reaching 79% and 84% in non-remitters and remitters, respectively.

#### Verbal fluency and working memory

**Fig. 3.**
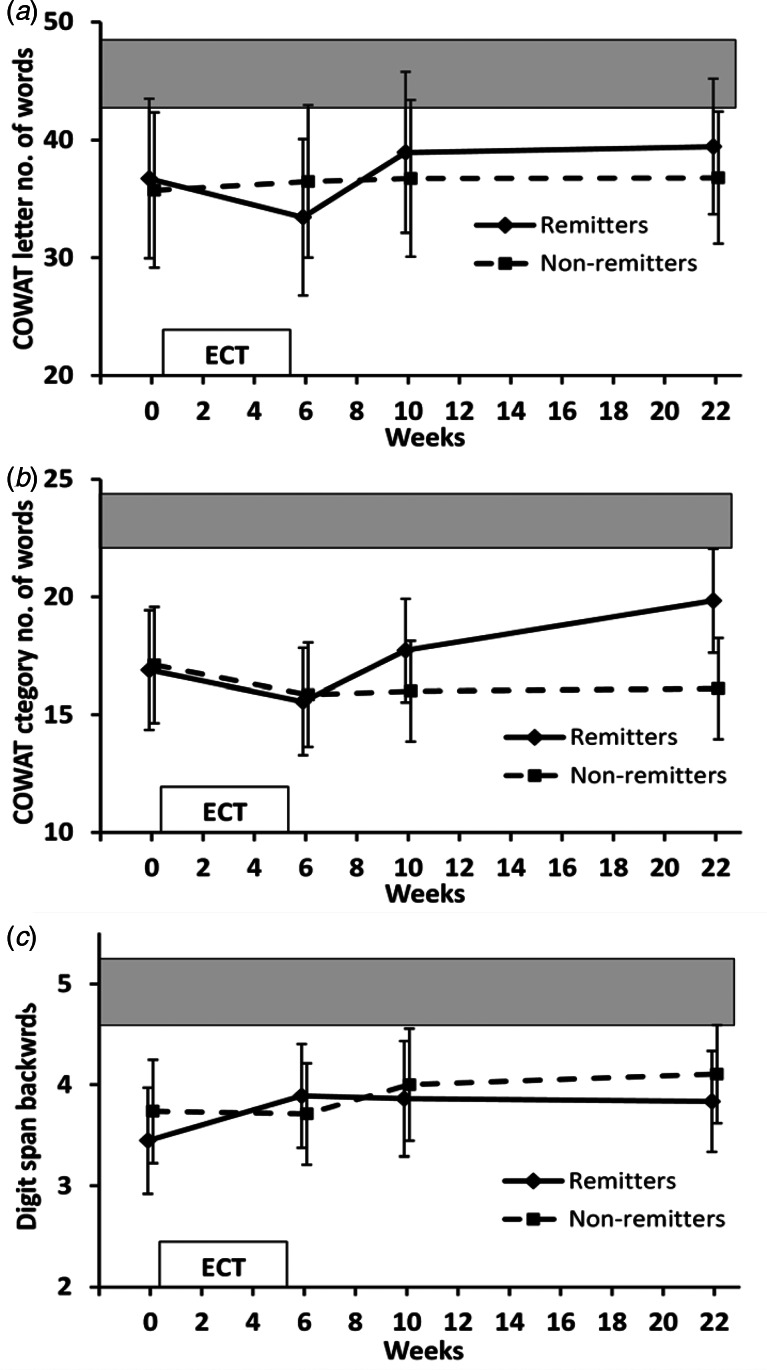
Executive function (COWAT letter and category fluency) and working memory (Digit span backwards) over time. Values are mean and 95% CI. Shaded area indicates 95% CI range for healthy controls. (*a*) COWAT letter fluency. ANOVA not significant. (*b*) COWAT category fluency. ANOVA time *p* = 0.014; time contrast between the end of ECT and 4-month follow-up *p* = 0.002. ANOVA group × time *p* = 0.011; group × time contrasts between baseline/end of ECT and 4-month follow-up *p* ⩽ 0.008. (*c*) Digit span backwards. ANOVA not significant.

COWAT letter fluency (Fig. 3*a*) showed no effect of time (*F*_3,105_ = 1.706, *p* = 0.18), group (*F*_1,35_ = 0.031, *p* = 0.86) or group × time interaction (*F*_3,105_ = 1.312, *p* = 0.28). At 4-month follow-up, both groups produced fewer words than healthy controls, significantly for non-remitters (*p* = 0.02) but not remitters (*p* = 0.19).

COWAT category fluency (Fig. 3*b*) changed significantly over time (*F*_3,105_ = 3.699, *p* = 0.014) with a significant group × time interaction (*F*_3,105_ = 3.874, *p* = 0.011). Remitters, but not non-remitters, showed an increase in the number of words produced from the end of ECT to final assessment. Time contrasts showed a trend decrease overall between baseline and end of ECT (*p* = 0.075) and a significant increase between the end of ECT and 4-month follow-up (*p* = 0.002). Remitters, compared with non-remitters, had a significant increase from both baseline and end of ECT to 4-month follow-up (*p* ⩽ 0.008). At final assessment however, both remitters and non-remitters still performed less well than healthy controls (*p* ⩽ 0.02).

Digit span backwards (Fig. 3*c*) showed a trend effect of time (*F*_3,105_ = 2.153, *p* = 0.098), but no effect of group (*F*_1,35_ = 0.189, *p* = 0.67) or group × time interaction (*F*_3,105_ = 0.872, *p* = 0.46). Time contrasts showed no significant change between baseline and end of ECT (*p* = 0.14) but a greater digit span at 4-month follow-up compared with baseline (*p* = 0.049) for all patients considered together. At 4-month follow-up, both remitters and non-remitters were still impaired compared with healthy controls (*p* ⩽ 0.05).

### Correlations

At 4-month follow-up, the GSE-My global memory correlated negatively with the MADRS score (*ρ* = −0.56, *p* < 0.001) and positively with the COWAT category fluency (*ρ* = 0.48, *p* = 0.003) and the AMI-SF percentage consistency (*ρ* = 0.34, *p* = 0.04).

## Discussion

The findings of this study are in partial agreement with our hypotheses with severely depressed patients who achieved remission 4 months after the end of a course of ECT having significant improvements in subjective memory, anterograde verbal memory and category (semantic) verbal fluency compared with those remaining depressed, who did not improve compared with before ECT. Mood status 4 months after ECT did not however influence anterograde visual memory, autobiographical memory, letter (phonemic) fluency or working memory. Compared to healthy controls, remitted patients remained impaired at 4-month follow-up with regard to working memory and category verbal fluency but did not differ significantly on anterograde verbal and visual memory and letter verbal fluency; however, the lack of repeat testing in controls limits interpretation as any improvement in performance due to practice effects in controls cannot be taken into account.

### Effect of ECT on cognitive function

At baseline, compared to healthy controls, depressed patients had impaired subjective memory and impaired performance on a range of neuropsychological tests of memory and executive function consistent with the literature (Pan et al., 2019). Following ECT, there was a significant decrease in scores for anterograde visual memory with the shape of the time-course (a dip after ECT with a subsequent improvement during follow-up) in keeping with a transient negative effect of ECT. This profile was also seen with verbal anterograde memory and category verbal fluency (although the decrease in scores after ECT was not significant), and autobiographical memory consistency improved after the end of ECT. These results are consistent with a transient effect of ECT on these measures as well, although the evidence is weaker. In contrast, ECT did not appear to affect subjective global memory, letter verbal fluency or working memory. While it is possible that we missed some of the effects of ECT, as testing occurred on average 4 days after the last ECT (Semkovska & McLoughlin, 2010), our findings are largely consistent with the meta-analysis by Semkovska and McLoughlin (2010) which found ECT impaired delayed verbal and visual recall and verbal fluency, with no significant effect on Digit span backward. Our findings for current global memory using the GSE-My are also similar to previous studies which have found either no effect of ECT (Brakemeier, Berman, Prudic, Zwillenberg, & Sackeim, 2011) or a modest impairment (Berman et al., 2008). Autobiographical memory measured using the AMI-SF is more difficult to interpret in the absence of normative data, and the decline following ECT is similar to that reported in the absence of ECT (Semkovska & McLoughlin, 2013). Nevertheless, relevant to our study, a randomised controlled trial in depressed bipolar patients using the AMI-SF found that immediately after ECT, there was a significantly greater reduction in memory consistency in those randomised to ECT than in those receiving pharmacotherapy only (73% *v.* 81%, *p* = 0.025) (Kessler et al., 2014), strikingly similar to the 75% consistency in our patients after ECT (Fig. 2*c*). Impairment of retrograde amnesia immediately after ECT is further supported by a study that showed a decline in a test of remote memory for events in the previous year after ECT that subsequently improved (Meeter, Murre, Janssen, Birkenhager, & van den Broek, 2011).

### Cognitive function four months after ECT

Studies examining cognitive function in the 3–6 months after ECT have varied both in the tests employed and as to whether or how they assess the influence of mood state. Excluding autobiographical memory, for which good evidence is lacking, there is a consistent finding that cognition is at least no worse than before treatment in the months after ECT, with some aspects improved although the exact picture differs between studies, and these are discussed below. For the GSE-My self-rated memory, the lack of overall change between baseline and the last follow-up masked a significant improvement in remitters compared with non-remitters. At 4-month follow-up, subjective memory correlated negatively with the mood in agreement with the literature (Vann & McCollum, 2019). For delayed verbal recall, the overall lack of significant change between baseline and 4-month follow-up also masked an improvement in remitters compared with no change in those still depressed. Previous studies predominantly report no overall improvement in anterograde verbal memory in the months after ECT similar to our results (Bosboom & Deijen, 2006; Mohn & Rund, 2016; Nuninga et al., 2018; Vasavada et al., 2017; Verwijk et al., 2014; Ziegelmayer et al., 2017), with one study finding an improvement (Bodnar et al., 2016), and another an improvement on one test and not in another (Obbels et al., 2018). Four studies examined a potential influence of mood and found no effect in contrast to our findings (Bosboom & Deijen, 2006; Nuninga et al., 2018; Vasavada et al., 2017; Ziegelmayer et al., 2017), but small sample sizes mean they lacked statistical power. Studies of category verbal fluency at medium term follow-up after ECT have found improvement (Mohn & Rund, 2016; Obbels et al., 2018) or no change (Bodnar et al., 2016; Nuninga et al., 2018) with one small study that examined the effect of mood finding no effect (Nuninga et al., 2018). Our results show both an overall improvement in category verbal fluency 4 months after and a clear effect of improvement with remission. For letter verbal fluency, we did not find any significant change, consistent with most (Bodnar et al., 2016; Nuninga et al., 2018; Obbels et al., 2018), but not all (Verwijk et al., 2014) studies, nor a difference between remitted and non-remitted patients.

Interpretation of our results for autobiographical memory is limited because of the AMI-SF scoring method (see Methods section). Of note consistency improved significantly after the end of ECT and was >80% when tested 5–6 months after baseline which is no worse than that reported in the literature for participants not receiving ECT (Bjoerke-Bertheussen et al., 2018; Semkovska & McLoughlin, 2013) suggesting a probable lack of impairment. This is also consistent with another study assessing remote memory for past events over the last year which found it had returned to at least baseline 3 months after ECT (Meeter et al., 2011). Nevertheless, the methodological difficulties in testing past memories experimentally make it difficult to exclude loss of specific past memories due to ECT (Fraser, O'Carroll, & Ebmeier, 2008).

Anterograde visual memory tested by the reproduction of a complex figure showed a different pattern to tests of verbal recall, with an improvement in scores from baseline to 4 months after ECT, and no evidence that it was influenced by the severity of depression. This is consistent with the improved scores found by most (Bodnar et al., 2016; Nuninga et al., 2018; Obbels et al., 2018), but not all (Vasavada et al., 2017), studies, of which none assessed the effect of mood state. The apparent improvement during follow-up is likely to be due to practice effects as discussed below. Finally, we found a possible slight improvement in Digit span backwards at final follow-up compared with baseline, but no effect of mood, suggesting that this measure is relatively insensitive to the current mood.

### Subjective and objective cognition after ECT

Subjective rating of memory after ECT is generally reported not to match the objective measurement on neuropsychological tests (Finnegan & McLoughlin, 2019) with the exception of global memory from one group using the self-rated GSE-My (Berman et al., 2008; Brakemeier et al., 2011). We also found significant positive correlations of the GSE-My current memory with category verbal fluency and, less strongly, with autobiographical memory consistency at 4-month follow-up. Of interest, both tests require the retrieval of information in long-term memory involving making conceptual or episodic associations. It is plausible that self-evaluation of global memory could be influenced by being aware of how well these two aspects of long-term memory are functioning. GSE-My global memory may therefore be a useful assessment of long-term memory in clinical practice. The utility of self-rated assessment of the effect of ECT is less clear given that it was consistently more negative that self-rated memory (Figs 1*a* and *b*) and neuropsychological task results. It is unclear the degree to which persisting depression after ECT could contribute to a negative evaluation of ECT's effect from our results. Other factors such as misattribution and anxiety may play a part (Finnegan & McLoughlin, 2019), and indeed we found a majority of patients had a negative expectation of the effect of ECT before treatment (Table 1). It has also been suggested that patients may be reporting subtle or patchy cognitive deficits not picked up by routine testing when reporting the effects of ECT (Finnegan & McLoughlin, 2019), but if so this is not reflected in self-evaluated current global memory.

### Comparison between patients and healthy controls

We only tested healthy controls on a single occasion limiting our ability to determine how cognitive function in a patient at follow-up compares with normal because of potential improvement due to practice effects in healthy subjects, for at least some tests (Nuninga et al., 2018; Obbels et al., 2018; Vasavada et al., 2017). Arguably, though, subjective memory is less likely to be affected in this way than neuropsychological tests. While we have some confidence in baseline comparisons between patients and controls, for follow-up assessments, this applies only when patients performed significantly worse than controls (category verbal fluency and working memory). In particular, the improvement we found during follow-up on anterograde visual memory tested is likely to be due to practice effects as two studies have reported improved performance on a delayed complex figure task in healthy controls when repeated (Nuninga et al., 2018; Vasavada et al., 2017), although whether practice effects occur to the same extent in ECT-treated depressed patients is uncertain. While medication effects cannot be excluded as a cause of impairment, our results are broadly consistent with the deficits reported more generally in patients remitted from depression (Semkovska et al., 2019).

### Strengths and limitations

This study contributes data to the important issue of cognitive functioning in the months after ECT. A strength of the study includes a prospective assessment of well-defined patient groups with a wide separation in depression severity at final follow-up, in the absence of systematic demographic or baseline differences. The low MADRS score in the remitted group indicates a high level of symptomatic recovery, similar to that seen in a community sample (Anderson et al., 2011). Important limitations include a secondary data analysis not planned in the original study design, with a relatively small sample size, which means the results need to be interpreted with caution. Included patients were required to have a reasonable cognitive function so it is not possible to extrapolate to cognitively impaired patients receiving ECT. There was a substantial drop out of patients by final follow-up making generalisation to the whole population uncertain, although at baseline, completers did not substantially differ from those dropping out (online Supplementary Table S1). The range of tasks was restricted to allow testing in a severely ill population, assessment of executive function was limited and we cannot comment on cognition beyond 4 months after ECT. We cannot exclude the potential adverse effects of medication on cognition in patients, but the similarity between the two patient groups makes this unlikely to explain the differences found between them. Healthy volunteers were only tested on one occasion so we lacked comparison data for autobiographical memory and did not have data on how performance might have improved due to practice effects for comparison with patients at follow-up.

## Conclusions

We found no evidence for persistent cognitive deficits occurring as a result of ECT and were able to provide evidence of the importance of remission on the degree of improvement of subjective memory and some aspects of neuropsychological test performance up to 4 months after ECT. For the latter, a benefit for remission was evident for anterograde verbal memory and category verbal fluency, which involve episodic and semantic verbal recall, respectively. We confirmed the importance of remission on improving self-reported memory measured by the GSE-My with preliminary evidence that this subjective measure is related to aspects of long-term memory performance, suggesting that it may be useful clinically to assess memory before and after ECT. Remitted patients 4 months after ECT still had cognitive deficits compared with healthy controls consistent with those reported in the literature. These deficits have been shown to contribute to functional impairment (Woo, Rosenblat, Kakar, Bahk, & McIntyre, 2016), suggesting that symptom relief alone is unlikely to restore full functional recovery in depressed patients.

## Supplementary material

For supplementary material accompanying this paper visit https://doi.org/10.1017/S0033291720000379. click here to view supplementary material
