# Supplementary material for: Cognitive function after electroconvulsive therapy for depression: relationship to clinical response
Source: Psychol Med. 2020 Feb 27;51(10):1647–56. doi: 10.1017/S0033291720000379 (PMC8327625; doi:10.1017/S0033291720000379)
Supplement: Supplementary file 1 [file S0033291720000379sup001.docx]

**Supplementary material**

**Cognitive function after electroconvulsive therapy for depression: relationship to clinical response**

Ian M Anderson MD, MRCP(UK), FRCPsych,^1*^ R Hamish McAllister-Williams PhD, MD, FRCPsych,^2^ Darragh Downey PhD,^3^ Rebecca Elliott PhD,^4^ Colleen Loo MD, FRANZCP^5^

1 Professor Emeritus of Psychiatry, Neuroscience and Psychiatry Unit, University of Manchester, Manchester Academic Health Science Centre, Manchester, UK

2 Professor of Affective Disorders, Newcastle University, and Honorary Consultant Psychiatrist, Cumbria, Northumberland Tyne and Wear NHS Foundation Trust, Newcastle upon Tyne, UK

3 Lecturer, Faculty of Biology, Medicine and Health, The University of Manchester, Manchester, UK

4 Professor of Neuropsychology, Neuroscience and Psychiatry Unit, University of Manchester, Manchester Academic Health Science Centre, Manchester, UK

5 Professor, School of Psychiatry, University of New South Wales, Black Dog Institute & St George Hospital, Sydney, Australia

*** Corresponding Author:**

Professor Ian M Anderson, Neuroscience and Psychiatry Unit, Division of Neuroscience and Experimental Psychology, University of Manchester, Manchester Academic Health Science Centre, Room G809, Stopford Building, Oxford Road, Manchester, M13 9PT, UK

Tel: +44 (0)161 275 7428

Email: [ian.anderson@manchester.ac.uk](mailto:ian.anderson@manchester.ac.uk)

This project was supported by the Efficacy and Mechanism Evaluation (EME) programme (reference number 10/90/04) funded by the Medical Research Council (MRC) and managed by the NIHR on behalf of the MRC–NIHR partnership.

**Summarised Trial Profile**

Patients randomised: **79**

Patients starting ECT: **72 (7 lost to follow-up after randomisation**: 4 didn’t start ECT for clinical reasons, 2 not included in the study for organisational reason, 1 medical contra-indication)

Patients completing post ECT assessment**: 60 (12 lost to follow-up after starting ECT**: 5 uncontactable, 2 withdrew consent, 2 detained under Mental Health Act, 2 discovered to have exclusion criteria not identified before recruitment, 1 lost capacity)

Patients completing one-month follow-up assessment: **48 (12 lost to follow-up after end of ECT:** 4 uncontactable, 4 withdrew consent, 3 further ECT, 1 organisational reason)

Patients completing 4-month follow-up assessment: **37 (11 lost to follow-up** **after one-month follow-up**: 5 uncontactable, 3 further ECT, 1 withdrew consent, 1 detained under Mental Health Act, 1 unavailable)

**Table S1:** Baseline comparison of study completers and non-completers (of patients who started ECT)

| **Measure** | **Non-Completers (N=35)** | **Completers (N=37)** |  |
| --- | --- | --- | --- |
|  | **Mean (95%CI)^1^** | **Mean (95%CI)^1^** | **p^2^** |
| Age | 55.6  (51.0 to 60.1) | 53.8  (50.1 to 57.6) | 0.56 |
| Sex, F:M | 23:12 | 23:14 | 0.75 |
| IQ | 107  (103 to 111) | 108  (104 to 111) | 0.89 |
| Years in FT education | 13.0  (11.9 to 14.1) | 14.3  (13.1 to 15.6) | 0.11 |
| MMSE | 28.9  (28.3 to 29.5) | 28.7  (28.2 to 29.3) | 0.70 |
| Diagnosis, UP:BP depression | 29:6 | 32:5 | 0.67 |
| Depression episode duration, months, Median [IQR] | 13  [6 to 37] | 9  [3.5 to 20] | 0.17 |
| MADRS | 32.8  (30.1 to 35.6) | 34.6  (31.9 to 37.2) | 0.35 |
| MGH Treatment resistance, Median [IQR] | 4.5  [2.5 – 5.5] | 3.5  [2.5 – 4.75] | 0.29 |
| Previous ECT, Yes:No | 16:19 | 19:18 | 0.63 |
| GSE-My Current global memory | 3.6  (3.1 to 4.0) | 3.7  (3.3 to 4.1) | 0.72 |
| GSE-My Expectation of ECT on memory, Negative:Nil/Positive^3^ | 14:20 | 25:11 | **0.02** |
| HVLT-R Delayed recall | 5.1  (4.0 to 6.3) | 6.8  (5.7 to 7.8) | **0.04** |
| MCGCFT Delayed recall | 16.5  (13.8 to 19.3) | 19.5  (17.2 to 21.7) | 0.09 |
| AMI-SF Baseline score | 43.8  (40.3 to 47.2) | 45.5  (42.3 to 48.7) | 0.47 |
| COWAT Letter fluency | 32.4  (27.4 to 37.4) | 36.2  (31.6 to 40.1) | 0.26 |
| COWAT Category fluency | 15.1  (13.1 to 17.1) | 17.0  (15.3 to 18.7) | 0.14 |
| Digit span Backward | 3.9  (3.5 to 4.4) | 3.6  (3.2 to 4.0) | 0.20 |

1 – unless otherwise stated; 2 - t-test/Chi^2^/Mann-Whitney U as applicable; 3- Two patients’ results missing

AMI-SF – Columbia Autobiographical Memory Interview-Short Form; BP – bipolar; COWAT – Controlled Oral Word Association Test; ECT – electroconvulsive therapy; FT – full-time; GSE-My – Global Self Evaluation of Memory; HVLT-R – Hopkins Verbal Learning Test-Revised; IQ – intelligence quotient; IQR – inter-quartile range; MADRS - Montgomery-Åsberg Depression Rating Scale; MCGCFT – Medical College of Georgia Complex Figure Test; MGH – Massechusetts General Hospital; UP – unipolar.
